# Transcriptional activity of transposable elements along an elevational gradient in *Arabidopsis arenosa*

**DOI:** 10.1186/s13100-021-00236-0

**Published:** 2021-02-27

**Authors:** Guillaume Wos, Rimjhim Roy Choudhury, Filip Kolář, Christian Parisod

**Affiliations:** 1 Department of Botany, Charles University, 128 01 Prague, Czech Republic; 2 Institute of Plant Sciences, University of Bern, 3013 Bern, Switzerland

**Keywords:** Alpine environment, *Arabidopsis arenosa*, Common garden experiment, Parallelism, RNA-seq, Transposable elements

## Abstract

**Background:**

Plant genomes can respond rapidly to environmental changes and transposable elements (TEs) arise as important drivers contributing to genome dynamics. Although some elements were reported to be induced by various abiotic or biotic factors, there is a lack of general understanding on how environment influences the activity and diversity of TEs. Here, we combined common garden experiment with short-read sequencing to investigate genomic abundance and expression of 2245 consensus TE sequences (containing retrotransposons and DNA transposons) in an alpine environment in *Arabidopsis arenosa*. To disentangle general trends from local differentiation, we leveraged four foothill-alpine population pairs from different mountain regions. Seeds of each of the eight populations were raised under four treatments that differed in temperature and irradiance, two factors varying with elevation. RNA-seq analysis was performed on leaves of young plants to test for the effect of elevation and subsequently of temperature and irradiance on expression of TE sequences.

**Results:**

Genomic abundance of the 2245 consensus TE sequences varied greatly between the mountain regions in line with neutral divergence among the regions, representing distinct genetic lineages of *A. arenosa*. Accounting for intraspecific variation in abundance, we found consistent transcriptomic response for some TE sequences across the different pairs of foothill-alpine populations suggesting parallelism in TE expression. In particular expression of retrotransposon LTR Copia (e.g. Ivana and Ale clades) and LTR Gypsy (e.g. Athila and CRM clades) but also non-LTR LINE or DNA transposon TIR MuDR consistently varied with elevation of origin. TE sequences responding specifically to temperature and irradiance belonged to the same classes as well as additional TE clades containing potentially stress-responsive elements (e.g. LTR Copia Sire and Tar, LTR Gypsy Reina).

**Conclusions:**

Our study demonstrated that the *A. arenosa* genome harbours a considerable diversity of TE sequences whose abundance and expression response varies across its native range. Some TE clades may contain transcriptionally active elements responding to a natural environmental gradient. This may further contribute to genetic variation between populations and may ultimately provide new regulatory mechanisms to face environmental challenges.

**Supplementary Information:**

The online version contains supplementary material available at 10.1186/s13100-021-00236-0.

## Background

The magnitude of genome evolution under environmental changes and underlying processes remain poorly known, despite a pressing need to understand mechanisms driving biodiversity in stressful conditions. Although regularly reported, the observation that genomes show high dynamics under the influence of stress-induced transposable elements (TEs) is among the most intriguing patterns [1–3]. As ubiquitous DNA fragments known for their ability to move from one location to another in the genome [4] of animals [5], plants [6] and bacteria [7], TEs were indeed considered as “parasitic DNA” using the host machinery for their replication [8]. Primarily, TEs induce (mostly deleterious) mutations and genome restructuring events, but their impact on adaptive processes remains hotly debated [1, 9]. Accordingly, how TEs are expressed and transpose in response to environmental challenges deserves further attention.

Early studies reported the induction of TE transcriptional and/or transpositional activity by biotic or abiotic stress [10]. Supporting evidence come from both class I (retrotransposons) or class II (transposons) TEs [11] otherwise distinguished by their transposition cycle using RNA or DNA intermediates (“copy-paste” or “cut-paste” mechanism), respectively [4]. In plants, a growing body of empirical studies reported specific activity of TEs in response to various stresses [12–14]. For instance, retrotransposons such as Tnt1 were induced by biotic stress in tobacco [15] or *ONSEN* by heat stress in *Arabidopsis thaliana* [16]. Similarly, DNA transposons such as mPing were demonstrated to be activated by irradiation in *Oryza sativa* [17] or Tam3 by low temperature in *Antirrhinum* [18]. Most studies have focused on a single TE exposed to one stressful condition, but only few studies have been conducted at a broader scale to study the influence of TEs in the adaptation of organisms to their natural habitats. For instance, variation in TE composition and/or in copy number was demonstrated in a natural population of wild barley in response to micro-climatic conditions [2] as well as among natural accessions of wild emmer wheat [19] or of *A. thaliana* [20, 21]. However, there is a lack of general understanding on how environment influences TE abundance and the activity of a large diversity of TEs.

Transcriptional activation being a crucial step towards possible transposition events supporting inheritable changes, transcriptome-wide surveys can therefore contribute highlighting necessary conditions under which TEs may impact genome evolution. However, recent transcriptomic analyses have reported global responses of the two main superfamilies of LTR retrotransposons (Copia and Gypsy) in *Arabidopsis spp.* after heat stress [22] and in *Eucalyptus* under environmental, particularly osmotic, stresses [23]. Such studies however remain scarce [23, 24] and the full hierarchy of TE clades nested within main TE types deserves further attention [25–27]. In particular, it remains unclear to what extent stressing conditions activate mostly random TEs through e.g. genome-wide epigenetic changes that would affect quiescent (i.e. functional but silent) copies depending on their location along chromosomes or rather would induce transcriptional activity of mostly closely-related TE copies spread along chromosomes but sharing specific regulatory motifs responding to corresponding environmental cues. Considerable advances taking variation within and among species into account appear necessary to shed firm light on responses of TEs to environmental factors and how this may contribute to the diversification of host genomes.

Our study system, *Arabidopsis arenosa*, is a close relative of *Arabidopsis lyrata* and *Arabidopsis thaliana*. It is a perennial plant encompassing diploid and autotetraploid populations across Central and Eastern Europe. Phylogeography and population genomics have been thoroughly investigated and it has been demonstrated that *A. arenosa* populations split into distinct genetic clusters corresponding to specific geographic regions [28]. In addition, a previous genome-wide study investigated dynamics of TEs following genome duplication and gave insight on how the genome of *A. arenosa* has been shaped by TEs [29]. The species occurs predominantly at low-elevation (up to ~ 1000 m a.s.l., termed ‘foothill ecotype’ hereafter) but scattered occurrences of *A. arenosa* in alpine stands above the timberline (~ 1500–2500 m a.s.l., ‘alpine ecotype’ hereafter) have been reported in several distinct mountain regions. The foothill and alpine ecotype, separated by a distribution gap of at least 500 m of altitude, are morphologically distinct, i.e. in height and floral traits [30]. The alpine ecotype had colonized the different mountain regions from the adjacent foothill ecotype and each foothill-alpine pair corresponds to distinct genetic clusters suggesting a parallel origin of the alpine ecotype [28, 30].

**Fig. 1:**
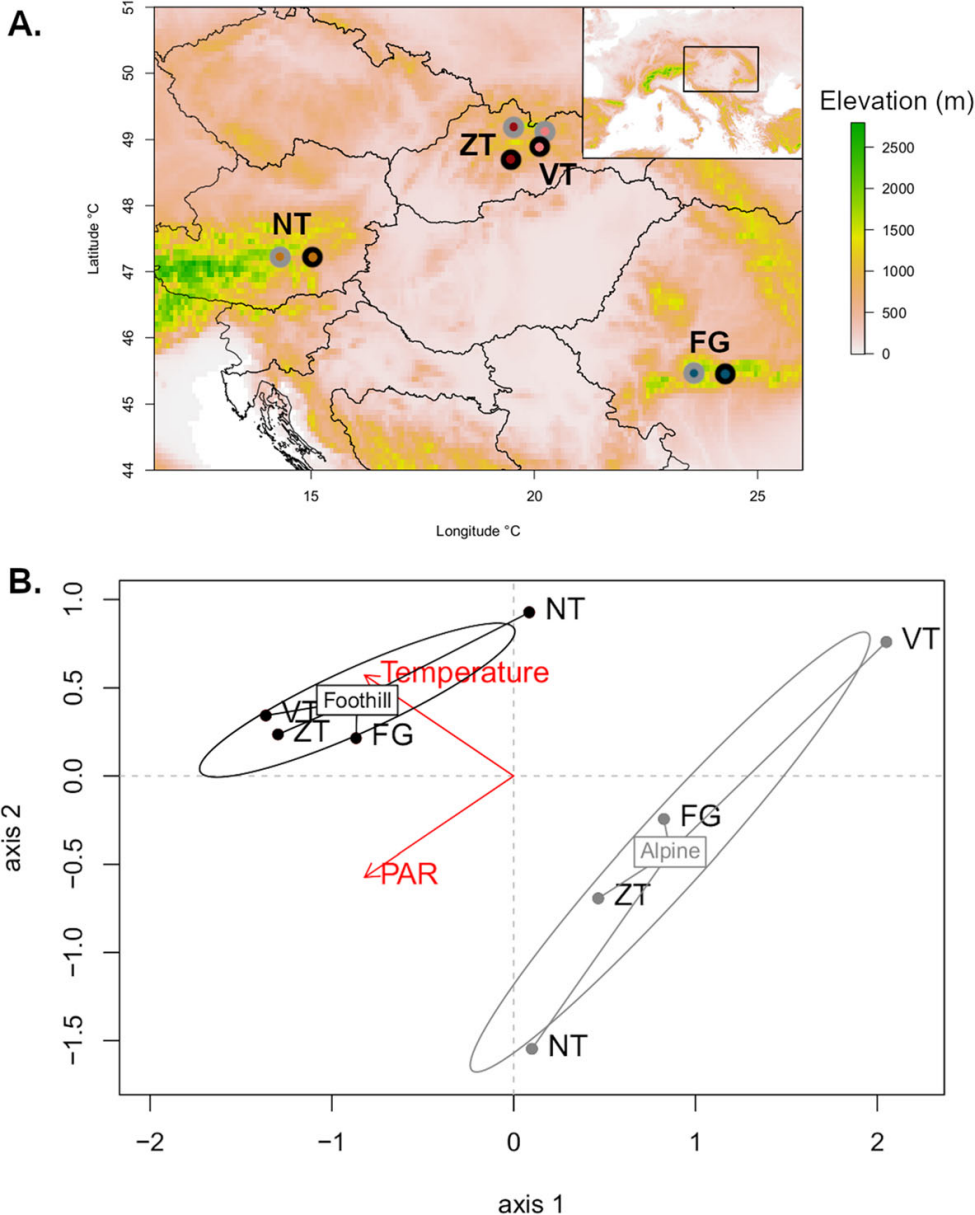
**a** Original locations of the eight populations from the four mountain regions used in this study. Dots coloured by ecotype (black = foothill, grey = alpine ecotype). **b** Principal component analysis based on the two environmental variables, temperature and irradiance, of the original sampling sites, coloured by ecotype (black = foothill, grey = alpine ecotype). We estimated the average values of temperature and irradiance (Photosynthetic Active Radiation [PAR]) over April, May and June that corresponds to the growth period of *A. arenosa*. The two variables were obtained from the high-resolution climate database SolarGIS, version 1.9, operated by GeoModel Solar (Bratislava, Slovakia). NT = Niedere Tauern (Austria), FG = Făgăraș (Romania), VT = Vysoké Tatry (Slovakia), ZT = Západné Tatry (Slovakia)

The elevation gradient constitutes an ideal system for investigating expression of TEs due to sharp environmental contrasts along short geographical distances depicting an environmental challenge typically faced by organisms. Here, we use RNA-seq, a widely used approach to study expression of TEs [31], to investigate natural variation in expression of a variety of TEs, with a particular focus on LTR Copia and LTR Gypsy clades, on four pairs of foothill and alpine populations (8 populations in total) from different mountain regions (‘region’, hereafter) within *Arabidopsis arenosa* (Fig. 1). Such replicated pairs of populations of foothill-alpine ecotypes involving genetically divergent lineages from across similarly differentiated environmental conditions are further leveraged to identify TEs repeatedly affected by corresponding environmental factors. Using 2245 consensus TE sequences (further classified into major classes and subclasses, see Methods section and Fig. S1) available from the closely related *Arabidopsis lyrata* (i.e. a highly similar genome to *A. arenosa* [29]), we first characterized the abundance of a comprehensive set of TEs among populations of *A. arenosa* and then used RNA-seq to assess their expression following growth under different treatments of temperature and irradiance (i.e. factors varying strongly along elevation gradients). We specifically addressed (1) to what extent the abundance and expression of consensus TE sequences varied between foothill and alpine ecotypes and across regions, (2) whether specific TE consensus sequences showed consistent differential expression among the four foothill-alpine pairs (suggesting parallelism in TE expression), and (3) what are the effects of particular experimental changes in temperature and irradiance on TE expression.

## Results

### Classification and abundance of consensus sequences in *A. arenosa* genome

We assessed genomic abundance of the major TE classes and subclasses across investigated regions and ecotypes of *A. arenosa* by mapping genomic reads of 71 individuals sequenced (8 populations, between 6 and 16 individuals per population) on the 2245 consensus TE sequences. We first looked at the difference in the number of reads aligned on each class (class I or class II elements) and subclass (LTR Copia and Gypsy, LINEs, SINEs and TRIM for class I and TIRs, MITEs and Helitrons for class II). We found a significantly higher number of reads aligned on class I than on class II (Fig. S2; Table 1A), with differences neither between regions nor ecotypes. Among the class I elements, we found a significant effect of region and ecotype on genomic abundance (with higher abundance in the foothill ecotype) but also of their interaction indicating some population-specific patterns (Fig. S2B). Similarly, genomic reads matching class II elements aligned mainly on TIR elements and showed different abundance among populations and ecotypes (higher abundance in the foothill ecotype) (Fig. S2C).

**Table 1:** Effect of class or subclass, ecotype and region on (A) genomic and (B) transcriptomic data

A) genome	All classes				Class I		Class II
**Variables**	**Df**_**variable/residual**_	***F***	**Variables**	**Df**_**variable/residual**_	***F***	**Df**_**variable/residual**_	***F***
Class	2/757	38.7***	Subclass	5/378	12172***	5/252	36849***
Ecotype	1/757	1.49	Ecotype	1/378	205***	1/252	38.9***
Region	3/757	1.01	Region	3/378	71.9***	3/252	155***
Ecotype*region	3/757	0.20	Ecotype*region	3/378	29.8***	3/252	11.4***
Class*ecotype	2/757	0.12	Subclass*ecotype	5/378	107***	5/252	6.11***
Class*region	6/757	0.29	Subclass*region	15/378	32.4***	15/252	11.4***
B) transcriptome					Class I		Class II
**Variables**	**Df**_**variable/residual**_	***F***	**Variables**	**Df**_**variable/residual**_	***F***	**Df**_**variable/residual**_	***F***
Class	2/1032	45.5***	Subclass	5/528	562***	5/352	930***
Ecotype	1/1032	0.73	Ecotype	1/528	1.07	1/352	3.37(*)
Region	3/1032	0.49	Region	3/528	9.34***	3/352	22.1***
Ecotype*region	3/1032	0.56	Ecotype*region	3/528	1.44	3/352	0.82
Class*ecotype	2/1032	0.86	Subclass*ecotype	5/528	9.37***	5/352	2.57(*)
Class*region	6/1032	3.30**	Subclass*region	15/528	11.0***	15/352	8.65***

Table shows the effect of class or subclass (tested separately on the class I and class II elements), ecotype, region and their interaction on the number of (A) genomic and (B) RNA-seq reads mapped on the 2245 consensus TE sequences. Table shows F-value, significance is indicated by *** *P* < 0.001, ** *P* < 0.01

**Fig. 2:**
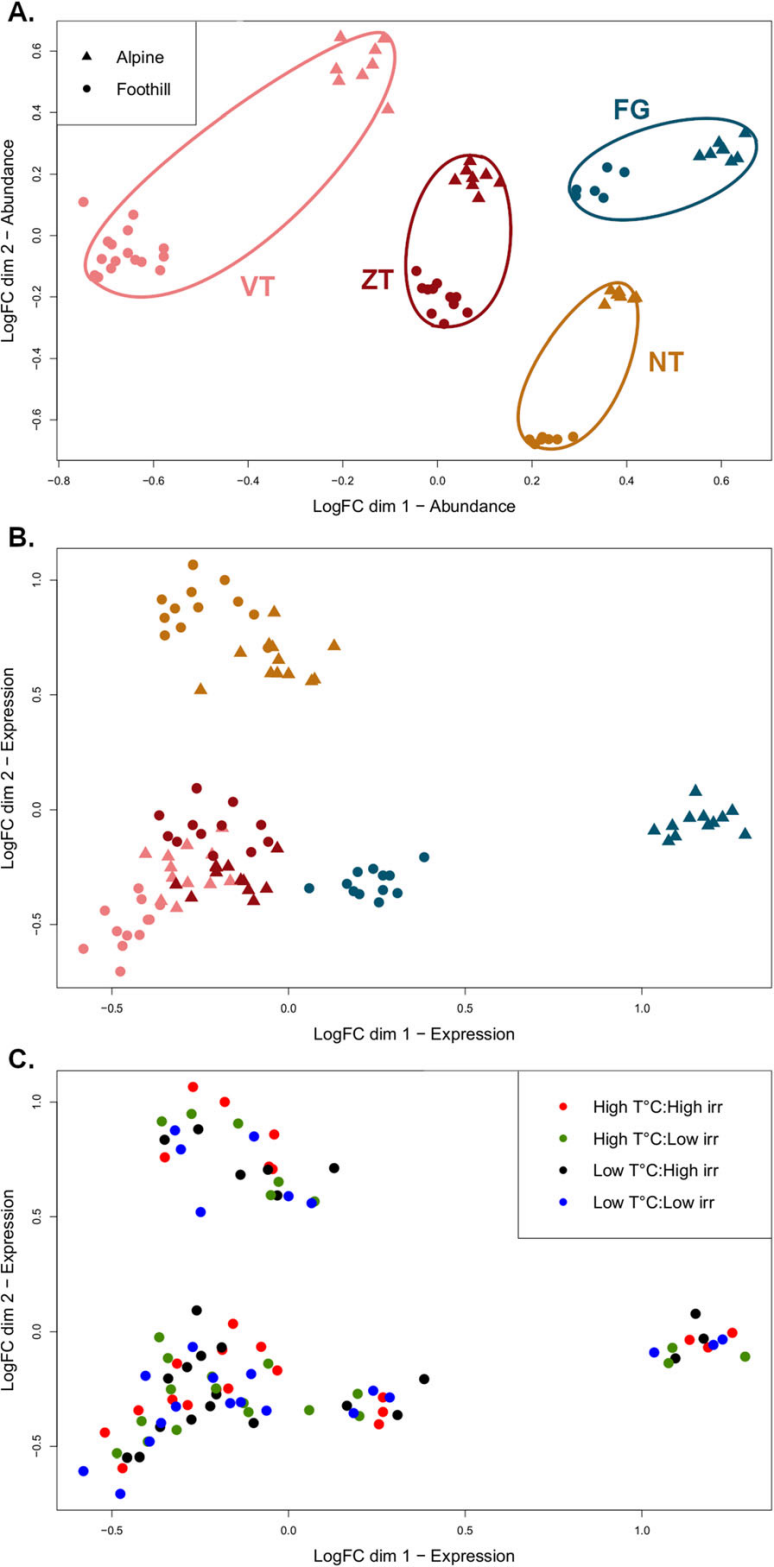
Multidimensional scaling plot showing the level of similarity in (**a**) genomic abundance (*N* = 71 individuals) and (**b**) and (**c**) in expression (*N* = 96 individuals) of the 2245 consensus sequences of *Arabidopsis arenosa* from studied populations. Each symbol represents one individual, in panels (**a**) and (**b**) triangles depict alpine and circles foothill ecotype and are coloured by region and in panel (**c**) symbols are coloured by treatment

We further investigated the overall level of similarity in TE abundance between the 71 individuals using multidimensional scaling plot (Fig. 2a). Consistently, individuals from the same population clustered together and region explained the greatest proportion of variance in TE abundance between individuals (PERMANOVA test; *R*^2^ = 69.7%, *F* = 153, *p* < 0.001), followed by ecotype (*R*^2^ = 12.7%, *F* = 84.0, *p <* 0.001). The interaction region*ecotype was also significant (*R*^2^ = 8.07%, *F* = 17.8, *p <* 0.001).

### General patterns of expression of consensus sequences

We assessed variation of expression of consensus sequences across regions and ecotypes using RNA-seq. We sequenced leaf transcriptome of three replicates for each region × ecotype × treatment combination for a total of 96 libraries and mapped RNA-seq reads on the 2245 consensus sequences for each individual.

To check whether higher expression of consensus TE sequences was due to their greater abundance in the genome, we first correlated expression (RNA-seq data) with abundance (genomic data) values for the consensus sequences differentially expressed between alpine and foothill ecotypes for each region (Table S1). We did not find any significant correlation between the expression and abundance values.

We found significant differences in the number of reads mapped on class I and class II elements as well as a regional effect (Table 1B). In general, more than half of the RNA-seq reads aligned on class II elements, except in FG region where more RNA-seq reads aligned on class I elements (Fig. S3A). Among the class I, the greatest proportion of RNA-seq reads aligned on non-LTR LINE and LTR Copia (Fig. S3B) with patterns of expression depending on the region of origin and ecotype (Table 1B). Among the class II, RNA-seq reads aligned especially on TIR sequences (Fig. S3C) and the number of RNA-seq reads aligned on each subclass varied also across regions.

Multidimensional scaling plot and PERMANOVA indicated that region, ecotype and treatment significantly affected overall TE expression of the 2245 sequences in *A. arenosa* (Fig. 2b and c). Region explained the greatest proportion of variance in TE expression between individuals (*R*^2^ = 41.4%, *F* = 35.8, *p <* 0.001) followed by ecotype (*R*^2^ = 6.49%, *F* = 16.8, *p <* 0.001) and treatment (*R*^2^ = 2.03%, *F* = 1.75, *p* = 0.025). Only the interaction ecotype*region was significant (*R*^2^ = 18.6%, *F* = 16.1, *p <* 0.001).

### Parallelism in the differential TE expression between foothill and alpine ecotypes

**Fig. 3:**
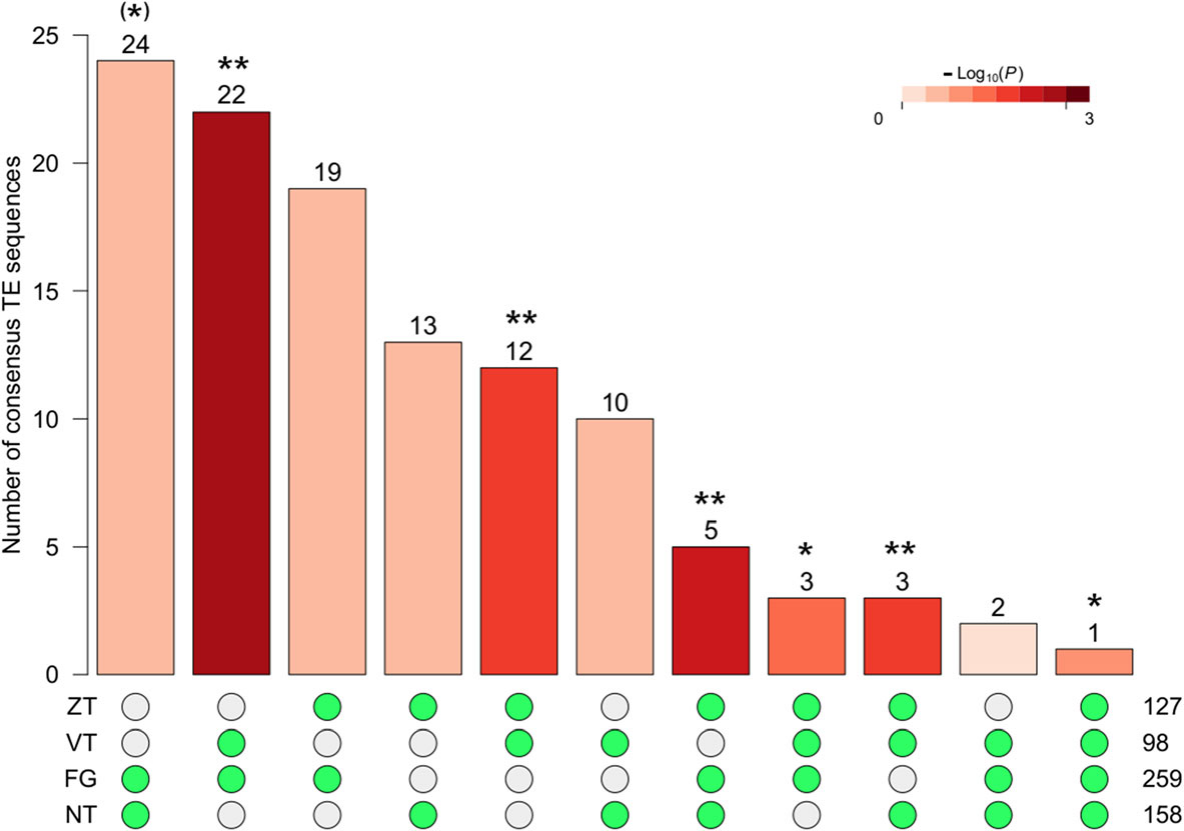
Number of consensus TE sequences differentially expressed between the foothill and alpine ecotype for each region and their overlap. Significance of each intersection tested by Fischer’s exact test is indicated by ** *P* < 0.01, * *P* < 0.05, ^(^*^)^ *P* < 0.1 and by different colour bars

To identify consensus TE sequences significantly affected by the alpine environment, for each region separately, we ran differential expression analysis between the foothill and alpine ecotype and overlapped the differentially expressed TEs across the four regions (Fig. 3, Table S2). Consensus TE sequences were considered as parallel if they were found to be differentially expressed in the same direction in at least two regions.

In total, we found 77 consensus TE sequences significantly differentially expressed between foothill and alpine ecotypes in the same direction across at least two regions (Table S3). Intersections across the four regions (1 consensus sequence) and across three regions, except for one, were significant (non-random overlap as determined by Fischer’s exact test), as well as some intersections across two regions indicating significant signs of parallelism in regard to TE expression (Fig. 3, Table S3). Among those, 35 TEs were class I elements, including 15 non-LTR LINE, 10 LTR Copia (particularly of the Ivana and Ale clades), and 7 LTR Gypsy (mostly from the Athila and CRM clades). The remaining class I elements were non-LTR SINE and TRIM. Forty class II TEs also showed such parallelism, including 29 TIR sequences of MuDR (14), CACTA (7), harbinger (3) and hAT (3). The remaining class II elements were Helitrons (10 sequences) and MITE (one sequence). Focusing on TEs showing the strongest evidence of parallelism of differential expression in all four regions, we observed only one consensus TE sequence, a non-LTR LINE element (‘RIX-incomp_MCL484_Alyr_TEdenovo-B-R2028-Map 3’), that showed consistently higher expression in the foothill ecotype. Up to 9 consensus TE sequences presented parallel expression changes across three regions, including five class I TEs such as LTR Copia (ALYCopia76 – Ivana clade) and LTR Gypsy (ATGP5A – CRM), and two class II TIR belonging to hAT (SIMPLEHAT2) and CACTA (EnSpm-6) that were more expressed in the foothill ecotype. In contrast, two non-LTR LINE, one SINE and two TIR elements (i.e. the CACTA EnSpm-6 and the harbinger HARB-4) were more expressed in the alpine ecotype.

We further analysed the main transcription factor-binding motifs located in the 77 consensus sequences to gain insight about potential factors controlling their expression (Table S4). We found that consensus TE sequences contained mainly motifs binding transcription factors involved in developmental processes (i.e. MADS-box, AP2, C2H2 or Dof) as well as hormone-responsive elements (i.e. auxin NAC and B3, and ethylene ERF). Specific motifs binding stress-responsive elements were identified and highlighted HSF (Heat transcription factor) in the consensus sequence of ALYCopia76 (LTR Copia – Ivana clade), GATA (light response) in 15 consensus TE sequences and FAR1 (light response) in one sequence.

### Effects of changes in temperature and irradiance on TE expression

We specifically assessed for the effects of changes in temperature and irradiance on the expression of consensus TE sequences to test whether these two abiotic factors that vary strongly with elevation may be potential drivers of TE differential expression between foothill and alpine ecotypes. For this, we performed treatment comparisons for each region separately. For the FG region, TE expression was found to be affected by neither temperature nor irradiance.

Effects of temperature were assessed by comparing the treatments: “High temperature: Low irradiance (Ht:Li)” and “Low temperature: Low irradiance (Lt:Li)” for the effects of temperature under low irradiance and “Ht:Hi” and “Lt:Hi” under high irradiance (Table S5). A total of 14 consensus TE sequences were significantly affected by temperature in individuals from the NT, VT and ZT regions. Ten of them were specifically more expressed under low temperature, including six LINE sequences, one LTR Copia (ALYCopia74 – Angela clade), one TIR MuDR-10, one MuDR-N17 and one TIR harbinger (harbinger-5). In contrast, four consensus TE sequences presented higher expression under high temperature, including two LINEs, one LTR Copia (ALYCopia94 – ivana clade) and one TIR MuDR-7.

We assessed effects of irradiance in a similar way by comparing “Low temperature: High irradiance (Lt:Hi)” and “Low temperature: Low irradiance (Lt:Li)” under low temperature and “Ht:Hi” and “Ht:Li” under high temperature. A total of nine consensus TE sequences were affected by irradiance among individuals from NT and ZT regions. Six presented higher expression under low irradiance, including three LINEs, one LTR Copia (ALYCopia52 – ale clade) and the TIRs MuDR-10 and MuDR-15, whereas three showed higher expression under high irradiance, including two class I LINE and one LTR Copia (ALYCopia76 – ivana clade).

We further compared consensus TE sequences specifically affected by temperature and irradiance with the 77 consensus TE sequences showing signs of parallelism previously identified (Table S3). We found that specific sequences overlapped and were affected by both elevation and temperature or irradiance: class I LINE and, as may be expected from its GATA and HSF binding motif, the LTR Copia AlyCopia76, but also class II TIR MuDR (MuDR-10).

To summarize, apart from differential expression between foothill and alpine ecotypes, we showed that some consensus TE sequences were also transcriptionally active in response to specific environmental factors typically varying along an elevation gradient.

### Expression of Copia and Gypsy clades

As LTRs are particularly active under stress in plants, we further focused on the effects of treatment, ecotype and region on the expression within each LTR Copia and Gypsy clade (Table S6). Analyses at the clade level confirmed the general trend we observed at the level of the consensus TE sequences, highlighting a minor effect of treatment but a strong regional effect, with or without the interaction with ecotype, on almost all TE clades. Only three clades were indeed significantly affected by treatments, including LTR Copia of the clade Ivana that also showed consensus TE sequences affected by temperature and irradiance and LTR Gypsy of the clades CRM and Tat.

Five clades were significantly affected by ecotype without the confounding effect of region: two LTR Copia, Sire (more expressed in alpine ecotype) and Tar (more expressed in in the foothill ecotype), and three LTR Gypsy, Athila, CRM and Reina (all more expressed in the foothill ecotype). Consensus sequences of LTR Copia from the clades Ale and Ivana were already identified as differentially expressed between the foothill and alpine ecotype, and here exhibited a significant ecotype*region effect. Similarly, consensus sequences of LTR Gypsy from the clades Athila and CRM clades were also differentially expressed between the two ecotypes and showed signs of parallelism.

## Discussion

Multiple pairs of foothill-alpine populations of *A. arenosa* from distinct mountain regions were here used to investigate how the environment interacts with the genomic abundance and expression of TE diversity both at a local scale and generally across a species.

Despite considerable intraspecific variation, we identified consensus TE sequences showing repeated transcriptional plasticity across regions indicating a common expression in response to specific environmental factors such as temperature and irradiance. Several differentially expressed TE sequences were closely related and formed specific clades showing detectable effects of ecotype or treatment within not only class I LTR Copia (e.g. Ivana and Ale clades) and LTR Gypsy (e.g. Athila and CRM clades) but also non-LTR LINE or class II TIR MuDR. Although TEs from some of these clades have been associated with stress in other species [14], our survey also highlighted new TE clades that potentially contain stress-responsive elements (e.g. LTR Copia Sire and Tar, LTR Gypsy Reina). Hence, such a transcriptome-wide survey contributes to shed further light on the plastic expression of TEs in response to environmental cues, but additional work is necessary to further delineate those TEs.

Our profiling of the abundance and expression of main clades within TE classes is generally matching the expected abundance of class I elements in plant genomes [10, 32] and the pervasive contribution of LTR Copia and LTR Gypsy as well as non-LTR LINEs and TIR to genomes in the *Arabidopsis* model [33]. Consistent with a previous study in *A. arenosa* [29], LTR Copia and non-LTR LINE for class I and TIR for class II contributed most to expression. Here, we further highlighted that differential expression was not significantly associated with the abundance of consensus TE sequences across samples, suggesting that abundant TEs may represent mostly silent remnants of past proliferation events, as shown in tomato for different Copia and Gypsy clades [34]. Accordingly, our survey captures specific transcriptional activity of particular TE clades despite the great variation among the different TEs and plant populations.

Our detailed investigation of differential TE expression between repeatedly evolved alpine vs. foothill ecotypes and its interaction with temperature and irradiance sheds light on the processes affecting genome evolution in contrasted environments. Consensus TE sequences belonging to LTR Copia, non-LTR LINE and TIR MuDR were here particularly differentiated among ecotypes and showed a significant parallelism across the four foothill-alpine pairs. In particular, main groups of LTR retrotransposons showed different transcriptional activity between foothill and alpine ecotype, but only LTR Copia and particularly Ale and Ivana clades responded specifically to temperature and irradiance. In contrast to LTR Gypsy that are mainly located in gene-poor, centromeric regions [35, 36], LTR Copia that are typically enriched in gene-rich regions [35] appear to present specific expression in response to environmental cues. Our observations generally match prior reports of LTR Copia being associated with stress-responsive genes [22, 37] or specifically responding to stresses such as high temperature [16, 38]. Among other retrotransposons appearing consistent with transcriptional activity under stress, LINE elements were here affected by all treatments and also showed strong signs of parallelism in the differentiation of foothill vs. alpine ecotypes. Not much is known about the non-LTR LINE elements in plants [27, 29, 39], a study demonstrated an association between LINE elements and abiotic-stress responsive genes in maize [40], several LINEs were shown to be differentially expressed after a heat stress in *A. lyrata and A. thaliana* [22] or to be the source of important phenotypic differentiation in oil palm [41]. Among class II TEs, TIR MuDR and particularly MuDR-10 were clearly affected by our treatments. TIR elements are possibly among the most active elements in *Arabidopsis* [21, 29, 42] and seem to accumulate in genic regions [35]. Although factors inducing their transcription remain to be clarified, they seem to be particularly expressed after a heat stress in *Arabidopsis* species [22] and to be responsive to UV light in maize [43]. Similarly, the expression of Helitrons appeared significantly differentiated by ecotype, recurrently across the regions. However, little is known about Helitrons that are generally found across the genome in *Arabidopsis*, with a tendency to insert close to other Helitrons and to frequently capture functional gene fragments [44–46]. Accordingly, Helitrons are not unlikely to capture stress responsive genes [47] that may explain detectable expression in response to elevation.

Expression of related TE copies (i.e. clades) in response to environmental conditions typical of sites at low vs high elevation was found in parallel across multiple regions. Even if the parallel consensus TE sequences represented a small portion of all the sequences investigated here, our results highlighted that some of them may have evolved specialized expression in response to environmental factors and could have contributed to genome diversity in natural populations.

Underpinnings of the close relationship between TEs and stress factors remain elusive despite detailed understanding of molecular mechanisms controlling TE activity [19]. Non-mutually exclusive hypotheses postulate either that genome-wide epigenetic changes induced by stressing conditions enable the activation of silent TE copies or that TEs co-opted particular motifs in their promotors and are activated by specific biotic and abiotic stimuli (e.g. regulatory U3 motifs in the LTR Tnt1A in tobacco [15]), or that TEs take advantage of regulatory mechanisms of nearby genes for their expression. In our study, the surmised location of stress-transcribed TEs across genic regions further suggests that specific TE copies may benefit from nearby genes to regulate their own expression. The identification of specific motifs binding transcription factors involved mainly in developmental processes and responses to environmental stresses may also partially explain differential expression of consensus TE sequences. However, in the absence of an appropriate reference genome of *A. arenosa* to assess the structure of TE copies and the genic environment of related copies, it remains difficult to conclude on the exact factors controlling their transcription. Whether TE expression is generally dependent on selected copies in specific genome regions or on specific regulatory features controlling TE transcription (e.g. Tnt1; [15]) requires further investigations.

## Conclusion

Our works demonstrated that the *A. arenosa* genome harbours a considerable diversity of TE sequences, being consistent with observations in other *Arabidopsis* species. We further concluded that some consensus TE sequences contained transcriptionally active elements (but their exact number remains to be determined) in response to conditions reflecting a natural environmental gradient. In particular, we observed that temperature and irradiance had significant effects on specific LTR Copia and Gypsy clades, either previously reported as stress-induced (e.g. LTR Copia Ivana or LTR Gypsy Athila and CRM) or here surmised as such for the first time (e.g. LTR Copia Sire and Tar or LTR Gypsy Reina). Importantly, the expression of several TE clades that are consistently differentiated in populations having repeatedly colonized a contrasting (alpine) environment suggests transcriptional adjustment to stressful environments. Future works will have to quantify how much differential TE expression along a natural gradient contributes to generating genetic variation between populations and ultimately to provide new regulatory mechanisms. Such knowledge would offer crucial insights on the potential chain of events linking TE dynamics and genome evolution in face of environmental challenges.

## Methods

### Plant material

We sampled *Arabidopsis arenosa,* a perennial outcrosser naturally occurring throughout low- to mid-elevations across Central Europe. We collected seeds from four distinct mountain regions (termed ‘region’): Niedere Tauern in the Austrian Alps (NT), Făgăraș Mountain in Southern Carpathians in Romania (FG) and Vysoké Tatry (VT) and Zapadné Tatry (ZT) Mountain in Western Carpathians in Slovakia (Fig. 1a). In all those mountains, *A. arenosa* also occurs at high-elevation above the timberline, separated from the major foothill population by a distribution gap of at least 500 m of altitude, forming a distinct alpine ecotype with morphological differences persisting after two generations in common garden conditions [30]. The VT region is occupied by diploids while the three other regions are occupied by autotetraploids [48].

For each region, we collected seeds from 10 maternal plants in one foothill (between 600 and 1000 m a.s.l) and one alpine (between 1700 and 2200 m a.s.l) population (termed ‘ecotype’), a total of 8 populations were sampled. Previous genetic investigations of our populations revealed that alpine stands in the NT, FG and ZT + VT regions have been colonized independently from their foothill counterparts, each foothill-alpine pair corresponding to a distinct genetic cluster [28, 30]. In case of ZT and VT, the cytotypes are genetically closely related [49], however, to take into account a potential effect of the ploidy level [29], we considered each region as a separate unit hereafter.

### Classification of TEs

We used TE library available for *Arabidopsis lyrata subsp. lyrata* [50], a close relative of *A. arenosa* sharing high similarity with the *A. arenosa* genome [29, 51]. Accordingly, we used the RepetDB database [52] containing the 112,563 copies of transposable elements (TEs) annotated in the *A. lyrata* genome and classified into 2408 consensus sequences representative of class I and class II TEs that are likely conserved among such closely related species [33] (consensus TE sequences in FASTA available at; http://urgi.versailles.inra.fr/repetdb/begin.do#search?taxonGroup=81972). After removal of 163 pseudogenes, we used the remaining 2245 consensus TE sequences as representatives of main orders within class I, including Long Terminal Repeats (LTR) retrotransposons and non-LTR retrotransposons (LINEs and SINEs), and within class II TEs, including Terminal Inverted Repeat (TIR) transposons and Miniature Inverted-repeat Transposable Elements (MITE) as well as Helitrons described based on their specific “rolling circle” mechanism of transposition (Table S7). Protein coding domains of LTR-retrotransposons were identified using DANTE in RepeatExplorer2 [53], which uses LASTAL for alignment against the REXdb [54], and further classified into main TE lineages (thereafter, clades) nested within Copia (i.e. Ale, Angela, Bianca, Ivanna, Sire, Tar and Tork) and within Gypsy (i.e. ATHILA, CRM, Reina, Tat and Tekay) [11, 54–56].

Classification of the 2245 consensus sequences revealed 958 class I TEs mostly belonging to LTR Copia (261 sequences), Gypsy (273 sequences) and non-LTR LINE (235 sequences) and 1287 class II TEs mainly classified as TIR (766 sequences), Helitron (341 sequences) and MITE (100 sequences) (Fig. S1).

### Genomic data

We used genome resequencing data of 71 individuals (between 6 and 16 individuals per population) from a previous study [28] (Table S8). Each individual DNA sample was sequenced on Illumina HiSeq 2500 (2 × 150 bp; minimum × 10 coverage). We used trimmomatic-0.36 [57] to remove adaptor sequences and low quality base pairs (< 15 PHRED quality score). Trimmed reads were aligned on the 2245 consensus TE sequences using HISAT2 2.1.0 [58] with the default parameters. We counted the number of reads mapped on each TE consensus sequence with featureCounts v1.6.3 [59] and kept the uniquely mapped reads to only consider reads that aligned on TE consensus sequences without ambiguity. In total, between 10 and 25% of the genomic reads aligned on the TE consensus sequences. Alignment of genomic reads on consensus TE sequences was further used to estimate relative abundance of consensus sequences among each other and to assess correlation with expression.

### Rearing conditions

We first raised one generation of the field-collected seeds in growth chambers under constant conditions (21/18 °C, 16/8 h day/night, light ~ 300 μmol m-2*s-1) in pots filled with a mixture of peat and sand (ratio 2:3) to reduce potential maternal effects. For each population, ~ 14 flowering plants were hand-pollinated by a mixture of pollen from the same population generating seed families comprising a variable mixture of full- and half-siblings.

Seeds of the next generation were used in this study and were raised under four experimental treatments that varied in temperature and irradiance, two environmental parameters typically associated with elevation and distinguishing our foothill and alpine populations (Fig. 1b). The four treatments were: “High temperature: High irradiance” (Ht:Hi); “High temperature: Low irradiance” (Ht:Li); “Low temperature: High irradiance” (Lt:Hi); “Low temperature: Low irradiance” (Lt:Li). In each treatment we used seeds from 8 populations × 3 seed families; for each population the same seed families were used across treatments. Treatments Ht:Li and Lt:Hi were used to mimic native conditions at low and high elevations respectively, whereas treatments Ht:Hi and Lt:Li were used to test for specific effects of temperature or irradiance on TE expression.

Seeds from each family were haphazardly selected and sown in individual pots for stratification in growth chambers for 1 week (4 °C, constant darkness). Seeds were then germinated at 9 h dark/15 h light (150 μmol m^− 2^ s^− 1^) with constant temperature (21 °C) and relative humidity (50%) for 20 days. After germination, seedlings were split and placed into four distinct growth chambers corresponding to the four treatments. In each chamber, the light:dark cycle was identical (16 h light/8 h dark) for the entire duration of the experiment; growth chambers are equipped with blue, red and far-red light panels (wavelengths of 447-448 nm/627 nm/725–728 nm respectively). Rearing conditions are described in Table S9. Briefly, after being placed into separate growth chambers, temperature was gradually changed to reach: 18 °C/13 °C day/night in high temperature treatments or 10 °C/4 °C day/night in low temperature treatments. We used photosynthetic photon flux density as a measure of irradiance with values ranging from 280 to 980 μmol m^− 2^ s^− 1^ during the day in high irradiance treatments or from 50 to 200 μmol m^− 2^ s^− 1^ during the day in low irradiance treatments. Temperatures reflect average values experienced by plants during their growth period in spring and early summer (average temperature measured in the Austrian Alps from April to June at 600 m = 18 °C, at 2000 m = 10 °C) and irradiance based on average values reported in a previous study [60]. Treatments were applied until plants reached the 14-leaf stage so that plant material for RNA-seq was collected on plants of similar developmental stage.

### Sample collection, RNA extraction and sequencing

For transcriptome analysis, we randomly selected one individual per maternal line and for each population (8 populations × 3 maternal lines × 1 individual × 4 treatments = 96 plants sequenced in total). After each plant reached the appropriate stage (i.e. 51 and 86 days after transfer to separate growth chambers depending on the treatment), we collected the seventh rosette leaf at a similar time point (between 11 a.m. and noon) and leaf samples were immediately snap-frozen in liquid nitrogen. None of the plants had flowered at the time of collection. Total RNA was extracted using the NucleoSpin miRNA kit including a DNase treatment step (Macherey-Nagel, Düren, Germany) according to the manufacturer’s instructions. We assessed the purity and quantity of RNA with a Nanodrop 2000 spectrophotometer (Thermo Scientific, Wilmington, DE, USA) and RNA integrity with Agilent 2100 bioanalyzer (Agilent Technologies, Palo Alto, CA, USA).

The sequencing library was prepared using the Illumina TruSeq Stranded mRNA Kit (Illumina Catalog # RS-122-9004DOC), with specific TruSeq adapters ligated on the cDNA for each individual. Individual sequencing was carried out on Illumina HiSeq 4000 (Illumina, San Diego, CA, USA) on four lanes (4 lanes × 24 individuals) using 150-bp paired-end reads. After sequencing, raw data were filtered to remove low-quality reads. Data are available at Sequence Read Archives (project ID PRJNA575330; [61]). Quality of each individual library was checked using the software fastqc (http://www.bioinformatics.babraham.ac.uk/projects/fastqc/). Overrepresented sequences corresponding to TruSeq adapters were trimmed using cutadapt [62]. Sequencing generated between 8 and 36 million reads per individual.

### Alignment and differential gene expression

In order to estimate expression of the main TE clades, trimmed RNA-seq reads were aligned on the *A. lyrata* TE consensus sequences using HISAT2 2.1.0 [58] with the default parameters. Between 1.45 and 8.19% of the RNA-seq reads aligned to the TE consensus sequences. The number of reads mapped on each TE consensus sequence was counted with featureCounts v1.6.3 [59], we kept only the uniquely mapped reads. Differential expression analysis was performed using the edgeR v3.12.0 package [63] in Rstudio [64]. We, first, scaled the library size (‘calcNormFactors’ function), estimated dispersion (“estimateDisp” function) and obtained, for each region and treatment, list of differentially expressed TE consensus sequences between the foothill and alpine ecotype (“glmFit” function).

For each region, we tested for the effects of ecotype by comparing differential expression between foothill and alpine populations. Because the effect of treatment was small compared to the effects of ecotype and region (see second paragraph of results part), for each region, we included individuals raised under the different treatments (*N* = 2 ecotypes × 4 treatments × 3 individuals = 24 individuals per region). Finally, for each region, the model included ‘treatment + ecotype’ as variables. *P* values were adjusted for multiple testing with the Benjamini and Hochberg False Discovery Rate (FDR) correction and TE consensus sequences were considered as differentially expressed if FDR < 0.05. Differential expression of consensus TE sequences was considered as parallel when reported in the same direction in at least two regions. We performed a transcription factor binding site enrichment analysis on TE consensus sequences showing parallelism using FIMO (only motifs with q-value < 0.01 were selected), which searches a set of sequences for occurrences of motifs with DNA-binding specificities [65] from a non-redundant set of *Arabidopsis thaliana* transcription factor binding motifs included in PlantTFDB 5.0 [66].

Effects of temperature or irradiance were tested by comparing treatments for each region separately (*N* = 2 treatments × 2 ecotypes × 3 individuals = 12 individuals per region). Effects of temperature was tested by comparing “Ht:Li” and “Lt:Li” (effects of temperature under low irradiance) and “Ht:Hi” and “Lt:Hi” (under high irradiance). Similarly effects of irradiance was tested by comparing “Lt:Hi” and “Lt:Li” (effects of irradiance under low temperature) and “Ht:Hi” and “Ht:Li” (under high temperature). Consensus sequences were considered as differentially expressed if FDR < 0.05.

### Statistical analysis

We used permutational multivariate analysis of variance (PERMANOVA) to test for overall differentiation among the individual genomic and transcriptomic profiles. We first created multidimensional scaling (MDS) plots using the ‘MDSplot’ function in edgeR v3.12.0 [63] and extracted the corresponding distance matrix. The distance matrix was then used to compute PERMANOVA test (adonis2 function, vegan package; [67], number of permutation = 10,000) in RStudio [64] using region, ecotype and their interaction (genomic data) or treatment, region, ecotype and their interaction (transcriptomic data) as predictors.

After scaling genomic or RNA-seq reads according to library size, we computed RPKM values (reads per kilobase per million mapped reads) using rpkm function in edgeR v3.12.0 [63]. We used RPKM values in anova to test for the effects of TE clades, region, ecotype and their interaction on the number reads mapped on the consensus TE sequences and to correlate expression and genomic abundance values using Pearson’s correlation coefficient. We performed Fischer’s exact test (SuperExactTest package; [68]) to test for significant intersection (*p* < 0.05) across the regions (= parallelism).

We tested specifically for the effects of treatment, region, ecotype and region*ecotype on expression of LTR Copia and Gypsy clades using ANOVA (basic R package). We run the analysis at the clade level, for this we summed RPKM values of each consensus TE sequence of the same clade to estimate the expression of each clade for each individual (*N* = 96 individuals).

## Supplementary Information

**Additional file 1: Fig. S1**. Number of consensus TE sequences (*N* = 2245) for each class I and class II subclass. **Fig. S2**. Proportion of genomic reads (*N* = 71 individuals) aligning on (A) class I and class II, (B) class I subclasses and (C) class II subclasses, for each region and ecotype. NT = Niedere Tauern (Austria), FG = Făgăraș (Romania), VT = Vysoké Tatry (Slovakia), ZT = Západné Tatry (Slovakia). VT region is diploid and the three others regions are tetraploid. **Fig. S3**. Proportion of RNAseq reads (*N* = 96 individuals) aligning on (A) class I and class II, (B) class I subclasses and (C) class II subclasses, for each region and ecotype. NT = Niedere Tauern (Austria), FG = Făgăraș (Romania), VT = Vysoké Tatry (Slovakia), ZT = Západné Tatry (Slovakia). VT region is diploid and the three others regions are tetraploid. **Table S1**. Pearson correlation coefficient between expression and abundance values. For each region, we correlated expression of the consensus sequences more expressed in foothill and alpine ecotype with abundance. Expression and abundance values were averaged across all individuals of the same population. NT = Niedere Tauern (Austria), FG = Făgăraș (Romania), VT = Vysoké Tatry (Slovakia), ZT = Západné Tatry (Slovakia). **Table S6.** Table shows the effect of treatment, ecotype, region and ecotype*region on expression of LTR Copia and Gypsy clades. Table shows F-value, significance is indicated by *** *P* < 0.001, ** *P* < 0.01, * *P* < 0.05, ^(^*^)^ *P* < 0.1.

**Additional file 2: Table S2**. List of consensus TE sequences differentially expressed between the foothill and alpine ecotype for each region.

**Additional file 3: Table S3**. Lists of differentially expressed sequences consistently more expressed in foothill or alpine ecotype across two, three or four regions.

**Additional file 4: Table S4**. Motifs analysis of the 77 consensus TE sequences showing parallelism.

**Additional file 5: Table S5**. Lists of consensus TE sequences affected by temperature and irradiance for each region.

**Additional file 6: Table S7**. Classification of the 2245 consensus TE sequences.

**Additional file 7: Table S8**. Genomic data used in this study.

**Additional file 8: Table S9**. Rearing conditions of *Arabidopsis arenosa*.

## Data Availability

RNA-seq datasets used in this study are available at Sequence Read Archives (project ID PRJNA575330 [61]; https://www.ncbi.nlm.nih.gov/sra/?term=PRJNA575330). Genomic data for five of the eight populations is included within the article (and its additional files) [28] (project ID PRJNA484107; https://www.ncbi.nlm.nih.gov/sra/?term=PRJNA484107). For genomic data of three remaining populations (Table S8): the datasets generated and/or analysed during the current study are not publicly available due to the fact that they are part of another research project that will be published in the future (under the accession number SRP233571 on SRA) but are available from the corresponding author on reasonable request.
