# Supplementary material for: Transcriptional activity of transposable elements along an elevational gradient in Arabidopsis arenosa
Source: Mob DNA. 2021 Feb 27;12:7. doi: 10.1186/s13100-021-00236-0 (PMC7916287; doi:10.1186/s13100-021-00236-0)
Supplement: Supplementary file 1 — Additional file 1: Fig. S1. Number of consensus TE sequences (N = 2245) for each class I and class II subclass. Fig. S2. Proportion of genomic reads (N = 71 individuals) aligning on (A) class I and class II, (B) class I subclasses and (C) class II subclasses, for each region and ecotype. NT = Niedere Tauern (Austria), FG = Făgăraș (Romania), VT = Vysoké Tatry (Slovakia), ZT = Západné Tatry (Slovakia). VT region is diploid and the three others regions are tetraploid. Fig. S3. Proportion of RNAseq reads (N = 96 individuals) aligning on (A) class I and class II, (B) class I subclasses and (C) class II subclasses, for each region and ecotype. NT = Niedere Tauern (Austria), FG = Făgăraș (Romania), VT = Vysoké Tatry (Slovakia), ZT = Západné Tatry (Slovakia). VT region is diploid and the three others regions are tetraploid. Table S1. Pearson correlation coefficient between expression and abundance values. For each region, we correlated expression of the consensus sequences more expressed in foothill and alpine ecotype with abundance. Expression and abundance values were averaged across all individuals of the same population. NT = Niedere Tauern (Austria), FG = Făgăraș (Romania), VT = Vysoké Tatry (Slovakia), ZT = Západné Tatry (Slovakia). Table S6. Table shows the effect of treatment, ecotype, region and ecotype*region on expression of LTR Copia and Gypsy clades. Table shows F-value, significance is indicated by *** P < 0.001, ** P < 0.01, * P < 0.05, (*) P < 0.1. [file 13100_2021_236_MOESM1_ESM.docx]

**Supplementary data**

**Separate files:**

**Table S2**. Consensus sequences differentially expressed between the foothill and alpine ecotype for each region.

**Table S3**. Lists of differentially expressed sequences consistently more expressed in foothill or alpine ecotype across two, three or four regions

**Table S4**. Analysis of sequence motifs

**Table S5**. Consensus TE sequences affected by temperature and irradiance

**Table S7**. Classification of the 2 245 consensus TE sequences.

**Table S8**. Rearing conditions of *Arabidopsis arenosa*.

**Table S9**. Data used for genomic analysis

**Fig. S1**. Number of consensus TE sequences (N = 2 245) for each class I and class II subclass.


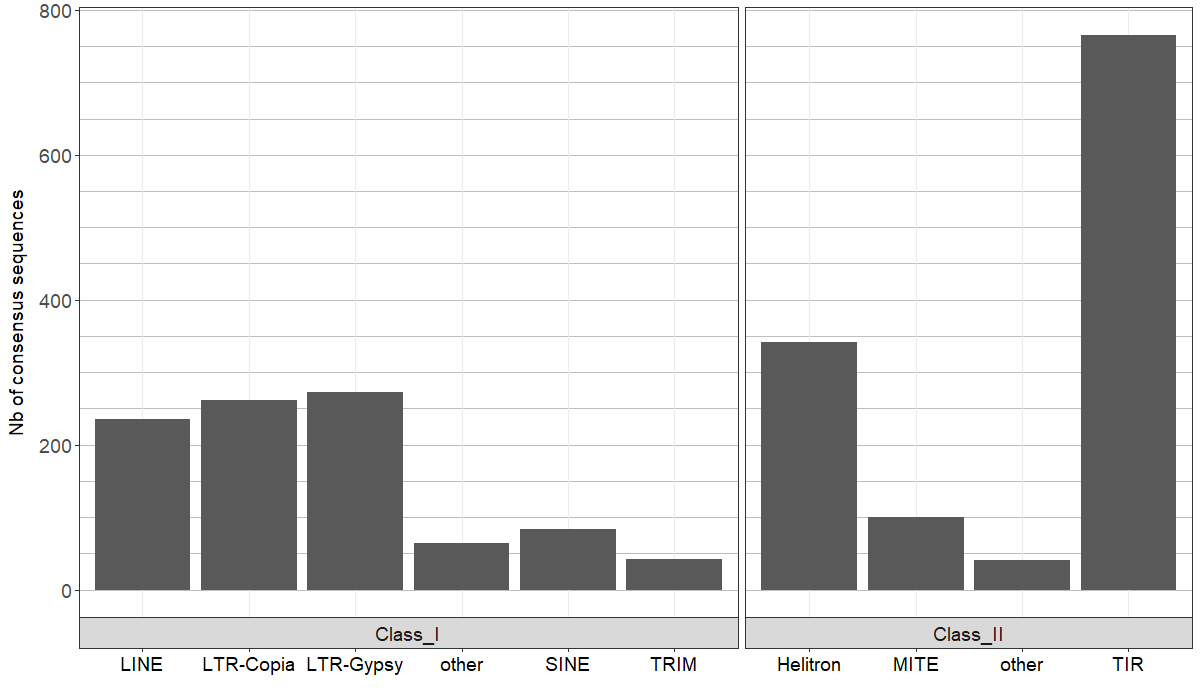


**Fig. S2**. Proportion of genomic reads (N = 71 individuals) aligning on (A) class I and class II, (B) class I subclasses and (C) class II subclasses, for each region and ecotype. NT = Niedere Tauern (Austria), FG = Făgăraș (Romania), VT = Vysoké Tatry (Slovakia), ZT = Západné Tatry (Slovakia). VT region is diploid and the three others regions are tetraploid.


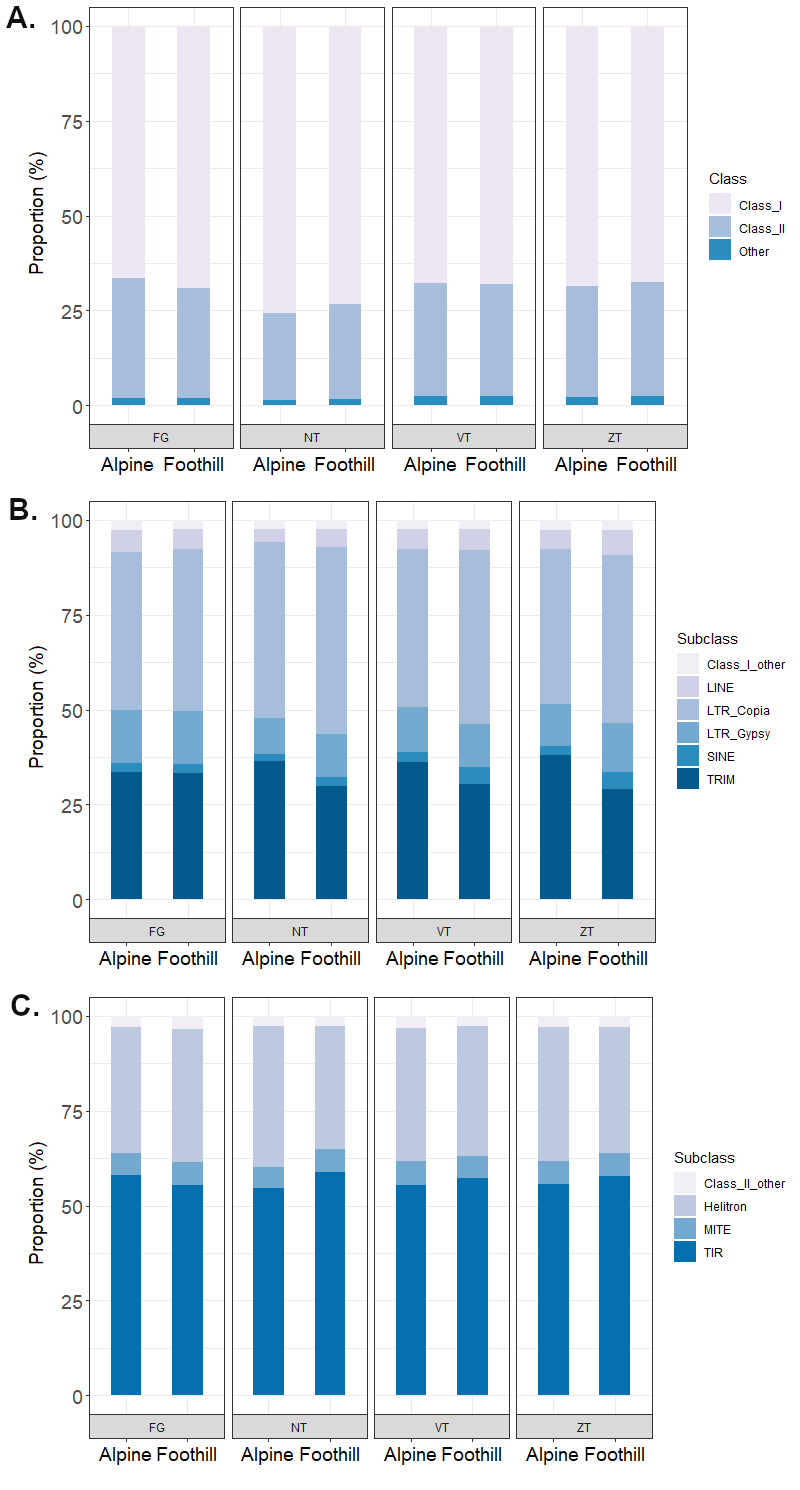


**Fig. S3**. Proportion of RNAseq reads (N = 96 individuals) aligning on (A) class I and class II, (B) class I subclasses and (C) class II subclasses, for each region and ecotype. NT = Niedere Tauern (Austria), FG = Făgăraș (Romania), VT = Vysoké Tatry (Slovakia), ZT = Západné Tatry (Slovakia). VT region is diploid and the three others regions are tetraploid.


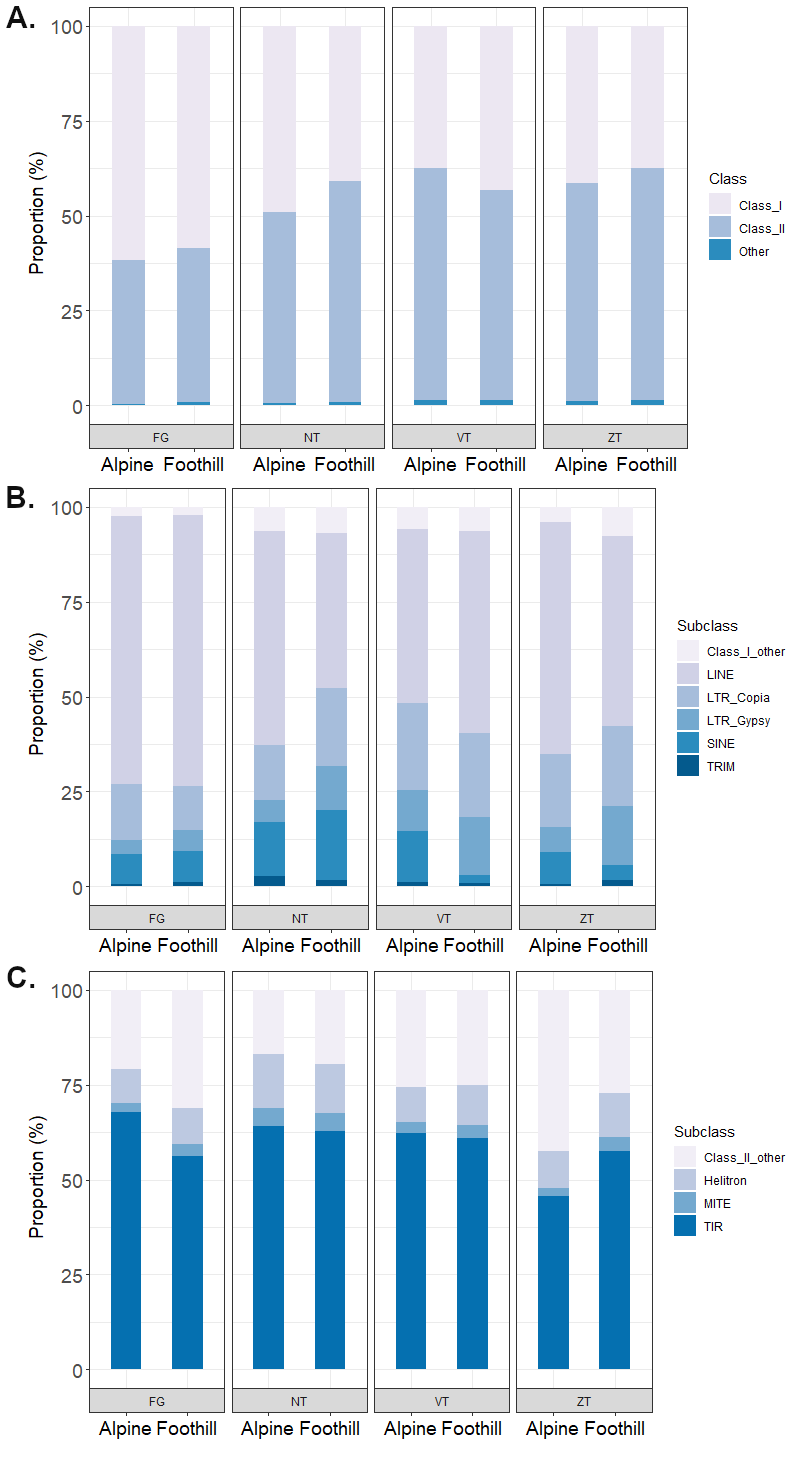


**Table S1**. Pearson correlation coefficient between expression and abundance values. For each region, we correlated expression of the consensus sequences more expressed in foothill and alpine ecotype with abundance. Expression and abundance values were averaged across all individuals of the same population. NT = Niedere Tauern (Austria), FG = Făgăraș (Romania), VT = Vysoké Tatry (Slovakia), ZT = Západné Tatry (Slovakia).

| Region | Population | More expressed in | df | coefficient | p-value |
| --- | --- | --- | --- | --- | --- |
| NT | AA255 | Foothill | 77 | 0.106 | 0.351 |
| NT | AA253 | Alpine | 77 | -0.22 | 0.051 |
| FG | AA067 | Foothill | 93 | -0.12 | 0.232 |
| FG | AA222 | Alpine | 162 | -0.09 | 0.245 |
| VT | AA016 | Foothill | 32 | 0.10 | 0.582 |
| VT | AA084 | Alpine | 62 | -0.12 | 0.330 |
| ZT | AA171 | Foothill | 58 | -0.21 | 0.100 |
| ZT | AA168 | Alpine | 65 | -0.24 | 0.051 |

|  |  | **LTR Copia** | | | | | | | | **LTR Gypsy** | | | | |
| --- | --- | --- | --- | --- | --- | --- | --- | --- | --- | --- | --- | --- | --- | --- |
|  | Df  _num/den_ | **Ale** | **Angela** | **Bianca** | **Ikeros** | **Ivana** | **Sire** | **Tar** | **Tork** | **Athila** | **CRM** | **Reina** | **Tat** | **Tekay Del** |
|  |  | *F* | *F* | *F* | *F* | *F* | *F* | *F* | *F* | *F* | *F* | *F* | *F* | *F* |
| **Treatment** | 3/85 | 0.74 | 0.70 | 0.75 | 1.10 | **4.56**** | 2.54(*) | 1.92 | 0.75 | 1.65 | **3.10*** | 0.97 | **4.11**** | 0.62 |
| **Region** | 3/85 | **15.7***** | 0.74 | **10.7***** | 0.74 | **12.2***** | **8.85***** | **10.3***** | **11.5***** | **3.31*** | 2.45(*) | **18.4***** | **8.55***** | 1.92 |
| **Ecotype** | 1/85 | 0.03 | 0.83 | 0.70 | 0.69 | 0.53 | **4.96*** | **9.07**** | 0.09 | **5.58*** | **8.92**** | **27.2***** | 1.86 | 0.05 |
| **Ecotype x region** | 3/85 | **5.35**** | **4.69**** | **9.11***** | 0.83 | **4.98**** | 0.63 | 2.09 | **8.98***** | **4.12**** | 2.13 | **7.03***** | 2.16 | **2.80*** |

**Table S6.** Table shows the effect of treatment, ecotype, region and ecotype*region on expression of LTR Copia and Gypsy clades. Table shows F-value, significance is indicated by *** *P* < 0.001, ** *P* < 0.01, * *P* < 0.05, ^(^*^)^ *P* < 0.1.
